# Suramin inhibits SARS-CoV-2 nucleocapsid phosphoprotein genome packaging function

**DOI:** 10.1016/j.virusres.2023.199221

**Published:** 2023-09-15

**Authors:** Irene Boniardi, Angela Corona, Jerome Basquin, Claire Basquin, Jessica Milia, István Nagy, Enzo Tramontano, Luca Zinzula

**Affiliations:** a Department of Molecular Structural Biology, Max Planck Institute of Biochemistry, Martinsried 82152, Germany; b Department of Life and Environmental Sciences, University of Cagliari, Monserrato 09042, Italy; c Department of Structural Cell Biology, Max Planck Institute of Biochemistry, Martinsried 82152, Germany; d Center of Research and Development, Eszterházy Károly Catholic University, Eger 3300, Hungary

**Keywords:** Antiviral agents, COVID-19, Nucleocapsid phosphoprotein, SARS-COV-2, Suramin, Viral replication

## Abstract

The coronavirus disease 2019 (COVID-19) pandemic is fading, however its etiologic agent severe acute respiratory syndrome coronavirus 2 (SARS-CoV-2) continues posing - despite the availability of licensed vaccines – a global health threat, due to the potential emergence of vaccine-resistant SARS-CoV-2 variants. This makes the development of new drugs against COVID-19 a persistent urgency and sets as research priority the validation of novel therapeutic targets within the SARS-CoV-2 proteome. Among these, a promising one is the SARS-CoV-2 nucleocapsid (N) phosphoprotein, a major structural component of the virion with indispensable role in packaging the viral genome into a ribonucleoprotein (RNP) complex, which also contributes to SARS-CoV-2 innate immune evasion by inhibiting the host cell type-I interferon (IFN-I) response. By combining miniaturized differential scanning fluorimetry with microscale thermophoresis, we found that the 100-year-old drug Suramin interacts with SARS-CoV-2 N-terminal domain (NTD) and C-terminal domain (CTD), thereby inhibiting their single-stranded RNA (ssRNA) binding function with low-micromolar K_d_ and IC_50_ values. Molecular docking suggests that Suramin interacts with basic NTD cleft and CTD dimer interface groove, highlighting three potentially druggable ssRNA binding sites. Electron microscopy shows that Suramin inhibits the formation *in vitro* of RNP complex-like condensates by SARS-CoV-2 N with a synthetic ssRNA. In a dose-dependent manner, Suramin also reduced SARS-CoV-2-induced cytopathic effect on Vero E6 and Calu-3 cells, partially reverting the SARS-CoV-2 N-inhibited IFN-I production in 293T cells. Our findings indicate that Suramin inhibits SARS-CoV-2 replication by hampering viral genome packaging, thereby representing a starting model for design of new COVID-19 antivirals.

## Highlights

• Suramin interacts with both the N-terminal domain (NTD) and C-terminal domain (CTD) of SARS-CoV-2 nucleocapsid phosphoprotein (N).
• Suramin prevents SARS-CoV-2 N ssRNA binding by docking to both the NTD RNA binding cleft and the groove at the CTD dimeric interface.
• Suramin inhibits SARS-CoV-2 N genome packaging *in vitro* by preventing formation of ribonucleoprotein (RNP) complex intermediates.
• Suramin reduces inhibition of type i interferon exerted by SARS-CoV-2 N.

## 1 Introduction

After more than three years since its emergence in Wuhan, capital of the Chinese Hubei province, the coronavirus disease 2019 (COVID-19) (Zhu et al., 2020a; Wu et al., 2020; Zhou et al., 2020a) caused by the severe acute respiratory syndrome coronavirus 2 (SARS-CoV-2) (Coronaviridae Study Group of the International Committee on Taxonomy of Viruses, 2020) has reached an estimated number of 676 million infected people, provoking more than 6.8 million deaths worldwide (COVID-19 Dashboard by the Center for Systems Science and Engineering (CSSE) at Johns Hopkins University (JHU), https://coronavirus.jhu.edu/map.html, last accessed 14rd March 2023). Moreover, the exceptionally rapid availability of licensed vaccines has significantly reduced hospitalization and fatal outcome (Soraci et al., 2022), yet the ongoing late pandemic waves still represent an international socio economic burden because of non-equitable access to those vaccines among countries, posing a threat to global health because of the recurrent infections from new, highly transmissible SARS-CoV-2 variants, and also due to the potential for more virulent and vaccine-resistant ones to rise (Nugent, 2022; Lino et al., 2022; Das et al., 2022). Likewise, the tremendous effort made to establish therapeutic countermeasures against COVID-19 yielded a portfolio of monoclonal antibody-based and small molecule-based drugs, yet those that were authorized thus far showed the limitations of being expensive, administrable only to hospitalized patients and targeted to only a few SARS-CoV-2 proteins, including the spike (S), the viral RNA-dependent RNA polymerase (RdRp) complex and the 3C-like or main protease (3CL-M^pro^) (Edwards et al., 2022; Robinson et al., 2022). In the face of such scenario, there is an urgent need to develop antiviral drugs against COVID-19 that are effective, safe and inexpensive. Towards this goal, an initial strategy has been consisting in exploring opportunities for targeted repurposing of approved drugs, in the idea that this may help reducing the costs and the time needed for preclinical research and clinical trials (Zhao et al., 2022). However, the *de novo* design of small molecule inhibitory compounds directly arising from the knowledge on SARS-CoV-2 antiviral targets still represents the ideal, albeit most difficult, strategy. Whether the strategy pursued, it is of utmost importance to acquire the most detailed information on both the molecular mechanisms and the atomic structure of as many as possible validatable targets within the SARS-CoV-2 proteome (Yan et al., 2022). Among these proteins, the nucleocapsid (N) phosphoprotein is a particularly attractive one, since it is a major structural component of the coronaviral virion, it exerts fundamental roles in the virus life cycle including packaging, transcription and replication of the viral genome, and is also a determinant of virulence and pathogenesis that contributes to the evasion of the host innate immune response by suppressing the production and the signaling of type I interferon (IFN-I) (Mu et al., 2020; Bai et al., 2021; Wu et al., 2023; Yu et al., 2023). Although the *N* gene is less prone to undergo mutations than other ones in the SARS-CoV-2 genome, it shows amino-acid changes consistent with positive selection for human-host adaptation (Jungreis et al., 2021). Nevertheless, its structural organization is highly conserved among SARS-related coronaviruses and follows a modular configuration where two globularly folded functional domains, namely the N-terminal domain (NTD) and the C-terminal domain (CTD), are interspersed with three intrinsically disordered regions (IDRs) termed as N-terminal arm (N-arm or IDR_1_), linker region (LKR or IDR_2_) and C-terminal tail (C-tail or IDR_3_), respectively (Chang et al., 2014). Furthermore, in regard with the main function exerted by N, namely its interaction with the single-stranded RNA (ssRNA) viral genome to form the ribonucleoprotein (RNP) complex, ssRNA binding is ascribed to both the NTD and CTD, whereas the sole CTD is responsible for the homo-oligomerization of the nucleocapsid protomers and the IDRs are involved in modulating both these activities (McBride et al., 2014; Chang et al., 2014). Thus far, several molecular architectures of the 419 amino acid-long, full-length SARS-CoV-2 N have been determined, including those by small-angle X-ray scattering (SAXS) for a dimeric protein either in the isolated form (Zeng et al., 2020) or in complex with the viral partner non-structural protein 3a (nsp3a) (Bessa et al., 2022), and by cryogenic electron microscopy (cryo-EM) for the RNP dimeric protomer complexed to a short tract of ssRNA (Zinzula et al., 2021). Likewise, a number of atomic structures of the two functional domains of SARS-CoV-2 N have been solved by several groups independently, including the one determined by NMR of an NTD complexed to a 10 bp-long ssRNA (Dinesh et al., 2020) and that of a high-resolution dimeric CTD determined by X-ray crystallography (Zinzula et al., 2021). In spite of differences in the amino acid sequence, these structures share the same overall folding with ortholog domains from other human (H) CoVs such as SARS-CoV (Chen et al., 2007; Saikatendu et al., 2007), Midde East respiratory syndrome (MERS)-CoV (Papageorgiou et al., 2016; Nguyen et al., 2019), HCoV-OC43 (Chen et al., 2013) and HCoV-NL63 (Szelazek et al., 2017), emphasizing the suitability of NTD and CTD as targets for developing broad spectrum drugs with pan-coronaviral valence. Noteworthy, NTD and CTD structures complexed to small-molecule ligands have been obtained for MERS-CoV (Lin et al., 2020; Hsu et al., 2022), HCoV-OC43 (Lin et al., 2014) and recently also for SARS-CoV-2 (Mercaldi et al., 2022), further highlighting the potential for targeting the genome packaging function of both domains. During the pandemic, as part of drug repurposing efforts aimed at identifying therapeutic solutions against COVID-19, one molecule that has drawn particular interest is Suramin, a drug developed a century ago for the treatment of African sleeping sickness and river blindness caused by trypanosomes and filarial parasites, respectively. As antiviral, Suramin was shown effective in inhibiting the replication *in vitro* of a wide variety of viruses, including human immunodeficiency virus, herpes simplex virus, hepatitis C virus, Dengue virus, enterovirus 71, norovirus (NoV), Chikungunya virus, Zika virus and Ebola virus (EBOV), only to mention some (Wiedemar et al., 2020). Moreover, regarding SARS-CoV-2 infection, Suramin was found able to interfere with early steps of the replication cycle (Salgado-Benvindo et al., 2020) and to specifically target the SARS-CoV-2 nsp12 subunit of the RdRp complex (Yin et al., 2021), the 3CL-M^pro^ (Zhu et al., 2020b; Eberle et al., 2021), the nsp13 helicase (Zeng et al., 2021) and, while this manuscript was in preparation, also the N protein (Mercaldi et al., 2022; Guo et al., 2023). Indeed, although Suramin is included in the World Health Organization (WHO) list of essential medicines (WHO, 2021), it cannot be considered a flawless therapeutic. In fact, Suramin suffers poor bioavailability due to its extensive negative charge, which, coupled to the high flexibility of its chemical structure, makes the molecule susceptible of promiscuous activity towards unspecific targets (Wiedemar et al., 2020; Dey et al., 2021). Furthermore, clinical studies have shown that Suramin may display several adverse side effects (Dey et al., 2021). Notwithstanding, the same chemical properties may turn advantageous in the context of designing target-guided, less toxic, and more selective derivatives based on its scaffold (Zeng et al., 2021), as recently shown by a computational framework that described the basis for Suramin binding promiscuity and its preferred interacting surfaces on proteins, also using the SARS-CoV-2 N NTD and RdRp complex as predictive benchmarks for the binding of Suramin analogs (Dey et al., 2021). Indeed, successful attempts to develop Suramin derivatives with lower cytotoxicity have been recently reported, and such molecules proven to be effective *in vitro* and *ex vivo* in animal models against tumor proliferation of metastatic cancer (Parveen et al., 2020; 2022) and cartilage degradation in chronic degenerative osteoarthritis (Green et al., 2023). For antiviral purpose, less cytotoxic Suramin analogs have been tested against NoV RdRp, also improving their bioavailability via cationic liposome formulations as delivery systems (Croci et al., 2014; Mastrangelo et al., 2014). Here, by combining results from biophysical, biochemical, computational, and biological experiments, we report that Suramin interacts with both SARS-CoV-2 N globular domains, inhibits their protein ssRNA binding function and thereby impedes packaging of the viral genome into an RNP complex. Our findings substantiate the potential of SARS-CoV-2 N as antiviral target, with a focus on the major role played by this protein during the virus life cycle, the nucleocapsid assembly. Moreover, we expand the current knowledge on SARS-CoV-2 proteins targeted by Suramin and its multifaceted inhibition of SARS-CoV-2 replication, also providing a novel framework for future rational design of Suramin analogs with high efficacy, low toxicity and specifically conceived to act as broad-spectrum inhibitors of coronaviral nucleocapsid proteins.

## 2 Materials and methods

### 2.1 Molecular cloning

SARS-CoV-2 N (GenBank: NC_045512.2) cDNA was obtained by synthetic preparation (BioCat) and cloned into pCDNA3.1+ (ThermoFisher Scientific) mammalian expression plasmid vector between restriction sites *Hind*III and *Xba*I, and into pET41b (Novagen) bacterial expression plasmid vector between restriction sites *Nde*I and *Xho*I. SARS-CoV-2 N CTD (residues 247–364) subcloning into pRSF-Duet (Novagen) plasmid bacterial expression vector was previously described (Zinzula et al., 2021). SARS-CoV-2 N NTD (residues 44–180) was subcloned into pRSF-Duet (Novagen) between the *Sac*I and *Avr*II restriction sites.

### 2.2 Protein expression and purification

SARS-CoV-2 N full-length, NTD and CTD recombinant hexahistidine (6xHis)-tagged proteins were produced as previously reported with minor modifications (Zinzula et al., 2021). Briefly, *E. coli* BL21 (DE3) (New England Biolabs) transformants were cultured in Terrific Broth (TB) medium supplemented with 50 mg mL^−1^ Kanamycin, in agitation at 220 rpm, 37 °C, up to 0.8 600 nm optical density (OD_600_), then induced for protein expression with 0.75 mM (full-length N and NTD) and 0.65 mM (CTD) isopropyl-b-d-1-thiogalactopyranoside (IPTG) overnight at 22 °C (full-length N) and 24 °C (NTD and CTD). Cell lysis was performed in buffer A (50 mM HEPES, pH 9.0 or pH 8.0; 500 mM NaCl; 6 % or 10 % (v/v) glycerol; 10 mM or 15 mM imidazole; for full-length N and CTD, respectively) or in buffer B for NTD (50 mM sodium phosphate, pH 8.0; 500 mM NaCl; 10 % (v/v) glycerol; 10 mM imidazole) all supplemented with 1 mg mL^−1^ Lysozyme (Sigma-Aldrich), 1 tablet 50 mL^−1^ cOmplete EDTA-free Protease Inhibitor Cocktail (Roche), ∼ 4000 Units *S. marcescens* Endonuclease (Max Planck Institute of Biochemistry Core facility) and 1 mM phenylmethylsulfonyl fluoride (PMSF). Lysates were sonicated and subjected to centrifugation at 32,000 x g for 35 min at 4 °C, and supernatant was collected and passed through 0.400 μm cutoff membrane filters (Merck MilliPore), then loaded on HisTrap FF crude columns (GE Healthcare) for purification by affinity chromatography. Only for full-length N, lysate was supplemented with 5 mM ethylenediamine tetra-acetic acid (EDTA), and supernatant was 6-fold diluted in buffer A to reduce EDTA concentration prior to column loading. Chromatographic resin beads were washed from non-specifically bound material in buffer C (25 mM or 50 mM HEPES, pH 9.0 or pH 8.0; 300 mM NaCl; 5 % (v/v) glycerol; 20 mM or 30 mM imidazole; for full-length N, NTD and CTD, respectively) and target proteins were eluted with 1 M (full-length N) or 600 mM (NTD and CTD) Imidazole. Full-length protein was dialyzed against buffer D (50 mM HEPES, pH 8.0; 100 mM NaCl), loaded on HiTrap ResourceQ column for anion exchange chromatography and eluted over a 0–100 % buffer E (50 mM HEPES, pH 8.0; 1.5 M NaCl) linear gradient. Pooled fractions from elution steps for each protein were loaded on a Superose 12 10/300 GL column (GE Healthcare) for size-exclusion chromatography (SEC) in buffer F (25 mM or 10 mM HEPES, pH 8.0 and pH 7.2; 100 mM or 150 mM NaCl; for full-length, NTD and CTD, respectively). Selected elution fractions from SEC were qualitatively assessed for purity and homogeneity by 4–12 % NuPAGE SDS-PAGE (ThermoFisher), then pooled, concentrated and used for subsequent experiments.

### 2.3 Miniaturized differential scanning fluorimetry (Nano-DSF)

SARS-CoV-2 N, NTD and CTD thermal unfolding profiles were acquired by measuring temperature-dependent shift in their intrinsic fluorescence at 330 nm (F330) and 350 nm (F350) emission wavelengths by using a Prometheus NT.48 (NanoTemper Technologies) instrument. Purified proteins were diluted in their storage buffer to 0.3 mg mL^−1^, 0.13 mg mL^−1^ and 0.25 mg mL^−1^, respectively, for the obtainment of optimal fluorescence counts, then loaded on standard capillaries (NanoTemper Technologies) and subjected to a 20–90 °C linear thermal gradient at 0.5 °C min^−1^ rate. Inflection points of fluorescence transition corresponding to melting temperature (Tm) values were determined as the first derivative maximum of the fluorescence intensity ratio at the measured wavelengths (F330/F350). For interaction between Suramin and the proteins, the Tm shift was assessed after addition to samples of 100 μM Suramin (Sigma-Aldrich) water solution and incubation for 1 hour at 25 °C. Suramin affinity to SARS-CoV-2 N NTD and CTD was assessed by titration of the compound over a 0.5–2000 μM concentration range against a fixed protein amount. For each experiment, data from at least three independent measurements were processed using the PR. ThermControl (NanoTemper Technologies) software.

### 2.4 Molecular docking

Suramin (PDB: 6CE2), SARS-CoV2-N NTD (PDB: 7ACT) and CTD (PDB: 6YUN) were processed for docking with program AutoDockTool (MGL-tools package, http://mgltools.scripps.edu/, last access on February 22, 2023) by the addition of polar hydrogens and partial charges on polar atoms, then saved as pdbqt format files. Atomic coordinates on docking targets for Suramin binding were used to build a discrete grid using AutoGrid (Goodford, 1985) software as the explored volume for searches with AutoDock Vina (Eberhardt et al., 2021) software. Following a knowledge-based approach from the literature, also driven by our experimental data, grid was centered near the groove of previously reported RNA binding sites on the two proteins, and box dimensions (54 × 42 × 42 Å^3^ and 54 × 52 × 56 Å^3^ for NTD and CTD, respectively) were chosen to allow free rotation of Suramin bonds in the source space. During computational analysis, proteins were constrained as rigid, whereas Suramin was left free to move. Docked posed listed in the AutoDock Vina docking search output as Suramin bound to the target proteins, were ranked based on their affinity as predicted binding free energy values (ΔG, kcal mol ^−1^), then selected for subsequent analysis.

### 2.5 Microscale thermophoresis (MST)

Purified SARS-CoV-2 N NTD, CTD, synthetic Cyanine 5 (Cy5)-ssRNA 7-mer (5′-Cy5-AUUAAAG-3′, Metabion) and Suramin were tested for interaction and inhibition of interaction using a Monolith NT.115 instrument (NanoTemper Technologies). Proteins and nucleic acid were diluted in MST buffer (buffer B supplemented with 0.05 % Tween-20) and, after mixing and incubation for 20 min at room temperature (RT), reaction samples were loaded into premium capillaries or standard ones (NanoTemper Technologies) for N NTD and CTD, respectively. For binding affinity assays, reactions were assembled at protein concentration range and ssRNA constant concentration of ∼ 0.0048–320 μM (N NTD) and 20 nM (Cy5-ssRNA 7-mer), and of ∼ 0.0048–160 μM (N CTD) and 40 nM (Cy5-ssRNA 7-mer). For Suramin inhibition of ssRNA binding, reactions were assembled at Suramin concentration range of ∼ 0.12–4000 μM and constant concentration of 160 μM (N NTD) and 20 nM (Cy5-ssRNA 7-mer), or at Suramin concentration range of ∼ 0.06–2000 μM and constant concentration of 160 μM (N CTD) and 40 nM (Cy5-ssRNA 7-mer). Measurement was taken at 23 °C temperature, 20 % LED-excitation power and high MST- power. For each MST trace corresponding to a given experimental condition, the measured relative fluorescence in the heated state (F_1_, *hot*, 1.5–2.5 s) was divided by the one measured in the cold state (F_0_, *cold*, −1–0 s) to obtain the corresponding normalized fluorescence (Fnorm) ratio. Dose-response MST curves were obtained by plotting Fnorm values expressed as part per thousand (‰) against ligand (*i.e.*, protein or Suramin) concentration. Data from at least three independent measurements representative of the MST trace fluorescence signal at 1.5 s (MST-On time) were analysed for dissociation constant (K_d_) and half-maximal inhibitory concentration (IC_50_) calculation using the MO. Affinity Analysis (NanoTemper Technologies) software.

### 2.6 Negative stain EM

SARS-CoV-2 RNP complex-like particles were reconstituted *in vitro* upon incubation of purified SARS-CoV-2 N with a synthetic ssRNA 56-mer (5′-AUUAAAGGUUUAUACCUUCCCAGGUAA CAAACCAACCAACUUUCGAUCUCUUGUAG-3′, Metabion) in a protein-to-RNA 4:1 molar ratio, overnight at RT in RNP buffer (10 mM HEPES pH 7,5; 150 mM NaCl; 0.5 mM MgCl_2_). The impact of Suramin on the formation of RNP complex-like particles was evaluated by supplementing the reaction with the compound at 50 μM final concentration. Purified SARS-CoV-2 N as negative control, and SARS-CoV-2 RNP complex-like samples, with and without Suramin, were diluted to 0.04 mg mL^−1^ and applied (10 μL) for 1 min to glow-discharged, carbon-coated 400 mesh Nickel grids (Electron Microscopy Sciences), then stained twice for 30 s with Methylamine Vanadate (Nano-Van, Nanoprobes) and Methylamine Tungstate (Nano-W, Nanoprobes) 1:1 ratio solution and air dried. EM data collection was performed on a Tecnai F20 (FEI) transmission electron microscope (TEM) equipped with a BM-Eagle 4 K CCD (FEI) camera, operating at 200 kV, 62,000 × nominal magnification corresponding to a 1.78 Å calibrated physical pixel size and applying a - 3.65 μm defocus target. Dataset were recorded by using SerialEM (Mastronarde, 2005) software and best micrographs for SARS-CoV-2 N (N = 30), SARS-CoV-2 *N* + ssRNA 56 bp (N = 11), and SARS-CoV-2 *N* + ssRNA 56 bp + 50 μM Suramin (N = 9) were chosen for random selection of 787, 784 and 659 representative particles, respectively. RNP-like complexes size was analysed by Fiji (Schindelin et al., 2012) software particle analysis tool by setting 5.5 nm as the minimum threshold for measurable radius, and particle radius distribution was analysed using Prism 9 v. 9.4.1 (GraphPad) software. Statistical significance was assessed with unpaired two-tailed *t*-test using Prism 9 v. 9.4.1 (GraphPad) software, with significance threshold set as *P* value < 0.05.

### 2.7 Cells and viruses

Vero E6-green fluorescent protein (GFP) cells (Janssen Pharmaceutical) were maintained in Dulbecco's modified Eagle's medium (DMEM, Gibco) supplemented with 10 % v/v fetal beef serum (FBS, Gibco), 0.075 % Sodium Bicarbonate (7.5 % solution, Gibco) and 1X Pen-strep (Euroclone) and kept under 5 % CO_2_ on 37 °C. SARS-CoV-2 (strain BetaCov/Belgium/GHB-03,021/2020) was provided by Katholieke Universiteit (KU) Leuven, Belgium. All virus-related work was carried out in certified, high-containment biosafety level-3 facilities at the University of Cagliari, Italy. Human embryonic kidney (HEK) 293T cells were grown in Dulbecco's modified Eagle's medium (DMEM; Gibco) supplemented with 10 % fetal bovine serum (FBS; Gibco) and 1 % penicillin/streptomycin (Sigma-Aldrich). Human Caucasian adenocarcinoma lung (Calu)−3 cells were maintained in DMEM (Gibco) supplemented with 10 % v/v FBS (Gibco), 0.075 % Sodium Bicarbonate (Gibco) and 1x Pen-strep (Euroclone). All cells were incubated at 37 °C in a humidified 5 % CO_2_ atmosphere.

### 2.8 SARS-CoV-2 replication assay

SARS-CoV-2 replication assay was performed as previously described (Pelliccia et al., 2022) by using a full replicant virus (SARS-CoV-2 strain BetaCov/Belgium/GHB-03,021/2020) on Vero E6-GFP cells. Cells were seeded at 10,000 cells/well in 96-well black cell-treated plates (PerkinElmer). The following day, cells were incubated with the control compounds at different concentrations and the virus at 0.01 multiplicity of infection (MOI). Compound was dissolved in 0.1 % dimethyl sulfoxide (DMSO). Seventy-two hours post infection the GPF signal, as direct index of cellular viability, was quantified by measuring the total-well fluorescence with a Victor 3 multiplate reader (PerkinElmer) set to excitation and emission wavelengths of 485 and 535 nm, respectively. The percentage of virus-induced cytopathic effect (CPE) was calculated considering the mock infected average (MIA) as 100 % of cell viability, and the readout from empty wells as blank, by using the formula: virus-induced CPE (%) = 100 – [(control infected sample – blank)/(MIA – blank) × 100]. The optimum of virus-induced CPE in control infected sample was set as ∼ 75 %, *i.e.*, leaving ∼ 25 % cells alive. For the determination of the compound half-maximal effective concentration (EC_50_), namely the one able to reduce by 50 % the virus-induced CPE, first the percentage of CPE was calculated considering the MIA as 100 % of cell viability, and the average of control infected samples (CIA) as baseline, by using the formula: residual CPE (%) = 100 – [(sample – CIA)/(MIA – CIA) × 100]. The values were then used to calculate the EC_50_ via non-linear regression by using on Prism 9 v. 9.4.1 software (GraphPad) the built-in function *dose-response inhibition,* log*-concentration-normalized response*. Experimental points represent the average and standard deviation of at least two sets of independent triplicates.

### 2.9 Reverse transcription-quantitative polymerase chain reaction (RT-qPCR) assay

For RT-qPCR assay, 2.4 × 10^5^ per well Calu-3 cells were seeded in transparent 12-well plates and incubated overnight. Cells were infected 24 h later with SARS-CoV-2 at 0.06 MOI in the presence of compound or 0,1 % DMSO (untreated controls). Cells were incubated for 1,5 h at 37 °C with 5 % CO_2_, then the virus was removed, replaced with complete medium with or without compound, and incubated at 37 °C with 5 % CO_2_. After 24 h cells were lysed in RLT buffer (Qiagen) and RNA extraction was performed using Tryzol (ThermoFisher Scientific) as per manufacturer instructions. One-step RT-qPCR was performed using 90 ng of RNA in 20 μL to quantify copy number of SARS-CoV-2 N target and glyceraldehyde-3-phosphate dehydrogenase (GAPDH) control genes, by using the primers 5′- GAGCTACCAGACGAATTCGTG-3′ (forward); 5′- CCTTCTGCGTAGAAGCCTTTTGG-3′ (reverse) and 5′-GAGTCAACGGATTTTGGTCGT-3′ (forward), 5′-TTGATTTTGGAGGGATCTCG-3′ (reverse), respectively. For detection, the Luna Universal One-Step RT-qPCR Kit (New England Biolabs) according to manufacturer's instructions and a CFX-96 RT-PCR system (Biorad), were used. As a control for stable gene expression in the sample, the threshold cycle (TC) value of the constitutively expressed GAPDH was determined. To determine SARS-CoV-2 *N* gene copy number, we considered the TC value of the PCR reaction negative control (TCneg) as baseline, by using the formula: *N* gene copy number = 2^(TCneg^ *^N^* ^gene – TC sample)^. To determine the EC_50_, namely the compound concentration able to reduce by 50 % the SARS-CoV-2- *N* gene transcription, the percentage of *N*-gene copy number was calculated considering the *N*-gene copy number of the infected sample as 100 %, and the one in the mock infected sample as baseline, using the formula: *N* gene copy number (%) = [(sample – mock infected)/(infected – mock infected) × 100]. The values were then used to calculate the EC_50_ via non-linear regression by using on Prism 9 v. 9.4.1 software (GraphPad) the built-in function *dose-response inhibition,* log*-concentration-normalized response*. Results report the mean and standard deviation of two independent experiments, each one in duplicate.

### 2.10 SARS-CoV-2 N IFN assays

SARS-CoV-2 N IFN assays were adapted from previously established protocols (Corona et al., 2022). Briefly, for the IFN-I promoter activation assay 1.5 × 10^4^ HEK 293T cells/well were seeded on a white 96-well plate, and 24 h later were co-transfected with 10 ng pRL-TK (Promega), 60 ng pGL-IFN-β-luc plasmid vectors, and 60 ng pCDNA3.1-SARS-CoV-2 N or pCDNA3.1 (empty) plasmid vector (of note, the amount of transfected DNA was chosen to keep the SARS-CoV-2 N inhibitory effect below 50 %). Cells were stimulated 24 h later cells with influenza A virus (IAV) RNA (Corona et al., 2022) and treated with Suramin, then pre-mixed with T-pro NTR III (T-Pro-Biotechnology) transfection reagent in reduced serum medium (Optimem, Gibco) according to the manufacturer's protocol. Cells were harvested 24 h later and treated as reported (Corona et al., 2022). For the IFN-stimulated response element (ISRE) promoter activation assay 1.5 × 10^4^ HEK 293T cells/well were seeded on a white 96-well plate, and 24 h later were co-transfected with 70 ng pISRE-luc, 10 ng pRL-TK (Promega) plasmid vectors, and 60 ng pCDNA3.1-SARS-CoV-2 N or pCDNA3.1 (empty) plasmid vector (of note, the amount transfected DNA was chosen to keep the SARS-CoV-2 N inhibitory effect below 50 %). Cells were treated 24 h later with 37 ng/mL IFN-α (Sigma-Aldrich) and Suramin (Sigma-Aldrich), then incubated for 8 h at 37 °C in 5 % CO_2_. Cells were harvested and treated as reported (Corona et al., 2022). Signal readout was Firefly/Renilla luciferase normalized, and the percentage was determined over stimulated pCDNA3.1+ (empty) plasmid vector control. Data were analysed with unpaired *t*-test using Prism 9 v. 9.4.1 (GraphPad) software, with significance threshold set as *P* value < 0.05.

### 2.11 Molecular graphics, structural and statistical analysis

Structural superposition rendering of SARS-CoV-2 NTD and CTD with Suramin and ssRNA were produced by using the PyMOL v.2.4 (http://www.pymol.org/) software. The output best poses from molecular docking were analyzed for interactions between residues and ligand by using the LIGPLOT (Wallace et al., 1995) software. Experimental data analysis and graph plotting were performed using the Prism v.9.4.1 (GraphPad) software. Data points are replicate averages with standard error (mean, SD) from at least three independent experiments.

## 3 Results and discussion

### 3.1 Suramin interacts with both functional domains of SARS-COV-2 N *in vitro*

**Fig. 1:**
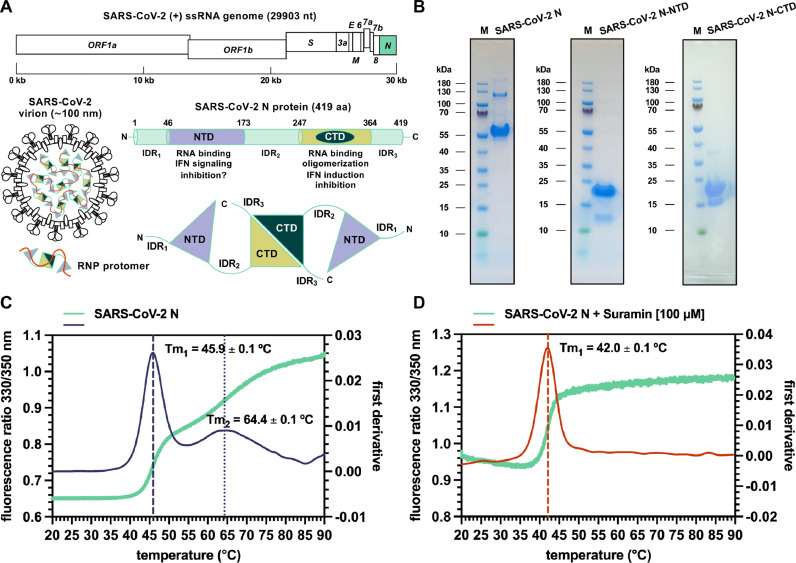
Suramin interaction with SARS-CoV2-N protein. (A) Schematic view of SARS-CoV-2 genome, virion, RNP protomer and structural organization of the N protein; the *N* gene is highlighted in lime green, NTD and CTD are depicted in blue bell and deep teal/wild willow green, RNA is depicted in pomegranate orange, respectively. (B) SDS-PAGE analysis of purified SARS-CoV-2 full-length N (left), NTD (middle) and CTD (right) (M, molecular weight marker). (C) Nano-DSF analysis of full-length SARS-CoV-2 N; thermal stability and conformational profile is shown by superimposed F330/350 ratio (lime green) and first derivative (deep-koamaru blue); conformational transitions corresponding to inflections in intrinsic fluorescence and related Tm_1_ (45.9 ± 0.1 °C) and Tm_2_ (64.4 ± 0.1 °C) are indicated as dashed and dotted lines, respectively. (D) Nano-DSF analysis of SARS-CoV-2 N in the presence of 100 μM Suramin; superimposed F330/350 ratio (lime green) and first derivative (pomegranate orange) are shown, with Tm_1_ (42.0 ± 0.1 °C; ∗∗∗∗ *P* < 0.0001) indicated as dashed line. Experimental points and Tm values are represented as averages and standard deviations from at least three independent experiments. Statistical analysis was performed by unpaired two-tailed Student's *t*-test (*N* = 2).

**Fig. 2:**
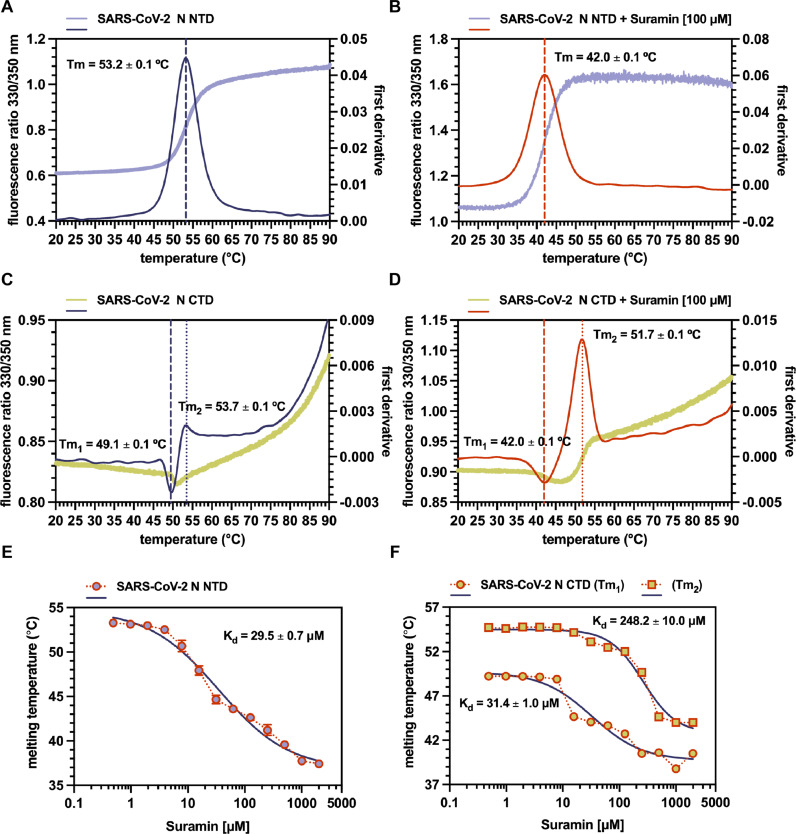
Suramin affinity to SARS-CoV-2 N NTD and CTD. (A) Nano-DSF thermal stability and conformational profile of SARS-CoV-2 N NTD; superimposed F330/350 ratio (blue bell) and first derivative (deep-koamaru blue), with conformational transition corresponding to intrinsic fluorescence inflection are shown; Tm (53.2 ± 0.1 °C) is indicated as dashed line. (B) SARS-CoV-2 N NTD Nano-DSF in the presence of 100 μM Suramin; superimposed F330/350 ratio (lime green) and first derivative (pomegranate orange) are shown, with shifted Tm (42.0 ± 0.1 °C; $\Delta T\approx 11^{\circ }C$; ∗∗∗∗ *P* < 0.001) indicated as dashed line. (C) Nano-DSF thermal stability and conformational profile of SARS-CoV-2 N CTD; superimposed F330/350 ratio (wild willow green) and first derivative (deep-koamaru blue), with conformational transitions corresponding to intrinsic fluorescence inflections are shown; Tm_1_ (49.1 ± 0.1 °C) and Tm_2_ (53.7 ± 0.1 °C) are indicated as dashed and dotted lines, respectively. (D) SARS-CoV-2 N CTD Nano-DSF in the presence of 100 μM Suramin; superimposed F330/350 ratio (wild willow green) and first derivative (pomegranate orange) are shown, with shifted Tm_1_ (42.0 ± 0.1 °C; $\Delta T\approx 7^{\circ }C$; ∗∗∗∗ *P* < 0.0001) and Tm_2_ (51.7 ± 0.1 °C; $\Delta T\approx 2^{\circ }C$; ∗∗∗∗ *P* < 0.0001) indicated as dashed and dotted lines, respectively. (E) Dose-response melting curve of SARS-CoV-2 N NTD in the presence of increasing amount of Suramin with apparent K_d_ (29.5 ± 0.7 μM) corresponding to ∼ 50 % of Tm decrease. (F) Dose-response melting curve of SARS-CoV-2 N CTD in the presence of increasing amount of Suramin with apparent K_d_ (248.5 ± 10.0 μM and 31.4 ± 1.0 μM) corresponding to ∼ 50 % of Tm_1_ and Tm_2_ decrease, respectively. Experimental points, Tm and K_d_ values are represented as averages and standard deviations from at least three independent experiments. Statistical analysis was performed by unpaired two-tailed Student's *t*-test (*N* = 2).

In the aim of finding repurposable therapeutic candidates that counter COVID-19 by targeting the SARS-CoV-2 replication cycle, we wanted to explore the druggability of SARS-CoV-2 N, towards either the protein-RNA interactions mediated by its NTD and CTD, or the protein-protein interactions mediated by its CTD, which together underlie the formation of SARS-CoV-2 RNP complex and nucleocapsid assembly (Fig. 1A). As experimental model we used a recombinant, bacterially expressed, full-length SARS-CoV N that we previously established (Zinzula et al., 2021) (Fig. 1B), and adopted the label-free Nano-DSF as a method to monitor changes in the protein tertiary structure and intrinsic-fluorescence emission during thermal denaturation (Vivoli et al., 2014). By means of this technique, is in fact possible to infer the interaction between a ligand and a given protein by simply measuring the shift in the Tm point of the latter upon its binding to the former (Baljinnyam et al., 2020). When tested as a control in the absence of compounds, the purified full-length recombinant SARS-CoV-2 N displayed two inflection points of its intrinsic fluorescence, corresponding to 45.9 ± 0.1 °C (Tm_1_) and 64.4 ± 0.1 °C (Tm_2_), respectively (Fig. 1C). In agreement with Nano-DSF data previously reported for the isolated CTD (Zinzula et al., 2021), the presence of two melting transition points is reminiscent of the dimeric nature of SARS-CoV-2 N in solution and may well correlate with different conformational changes undergone by the CTD during thermal gradient, likely consisting in an initial destabilization of the dimer followed by an unfolding of the monomers (Zeng et al., 2020). Next, we screened Suramin among other small-molecule compounds from an in-house library to assess its interaction with SARS-CoV-2 N. As shown, in the presence of Suramin at 100 μM final concentration SARS-CoV-2 N displayed only one inflection point (Tm_1_), whose value was lowered by almost four degrees (42.0 ± 0.1 °C, *P* < 0.0001) with respect to control (Fig. 1D and Supplementary Table 1). A possible mechanistic interpretation of this result, is that binding of Suramin to SARS-CoV-2 N destabilizes the protein tertiary structure and increases its flexibility, thereby anticipating the occurrence of conformational changes upon temperature increase. Therefore, given the presence in the full-length protein of the three highly flexible IDRs, we evaluated whether interaction with Suramin would specifically involve any of the two structured portions of SARS-CoV-2 N, and to this aim we sought to test by Nano-DSF the isolated SARS-CoV-2 N NTD (residues 44–180) and CTD (residues 247–364), that we expressed and purified as recombinant constructs (Fig. 1B). As shown, SARS-CoV-2 N NTD displayed a single inflection point (Tm_1_, 53.2 ± 0.1 °C) (Fig. 2A), whose value decreased to 42.0 ± 0.1 °C (*P* < 0.0001) in the presence of 100 μM Suramin (Fig. 2B and Supplementary Table 1). Similarly, the two inflection points displayed by SARS-CoV-2 N CTD (Tm_1_, 49.1 ± 0.1 °C; Tm_2_, 53.7 ± 0.1 °C) (Fig. 2C) were lowered in the presence of 100 μM Suramin to 42.0 ± 0.1 °C (Tm_1_, *P* < 0.0001) and 51.7 ± 0.1 °C (Tm_2_, *P* < 0.0001), respectively (Fig. 2D and Supplementary Table 1). Next, a titration of Suramin against fixed concentrations of SARS-CoV-2 N NTD and CTD was performed to quantitatively characterize the interaction in terms of measurable affinity of the compound for the two protein domains. As shown, Suramin decreased the Tm of SARS-CoV-2 N NTD in a dose-dependent manner over a 0.5–2000 μM concentration range, displaying an apparent K_d_ value of 29.5 ± 0.7 μM (Fig. 2E and Supplementary Table 2), which is consistent, albeit higher, with recently reported data from biolayer interferometry (Guo et al., 2023). Similarly, the two melting points of SARS-CoV-2 N CTD were lowered by Suramin in a dose-dependent manner over the same titration range (0.5–2000 μM), displaying apparent K_d_ values of 31.4 ± 1.0 μM and of 248.2 ± 10.0 μM for the Tm_1_ and Tm_2_ curves, respectively (Fig. 2F and Supplementary Table 2). Taken together, these results demonstrate that Suramin binds to both globular regions of SARS-CoV-2 N, with affinities in the low micro molarity range. Moreover, data showed that Suramin binding to NTD and CTD negatively affects the thermal stability of the two domains, introducing structural perturbations that go well beyond the local level, and this led us to the hypothesis that interaction with Suramin could result in a compromission of the functionality of the two domains.

### 3.2 Suramin docks to the SARS-COV-2 N NTD ssRNA-binding cleft and the basic groove at CTD dimer interface *in silico*

**Fig. 3:**
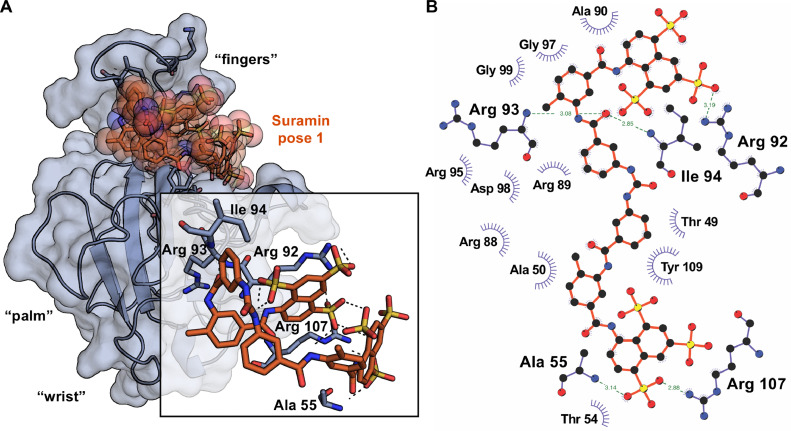
Model and interaction map for Suramin binding to SARS-CoV-2 N NTD. Cartoon 3D representation of Suramin putative binding mode to SARS-CoV-2 N NTD, according to best pose by molecular docking; predicted 3D-intermolecular interactions at binding site are zoomed in the inset. (B) 2D-diagram of predicted Suramin-residue interactions within the N NTD binding pocket; H-bonds and hydrophobic contacts are shown as dashed lines with distances between atoms indicated in angstrom and as arcs with spokes, respectively.

**Fig. 4:**
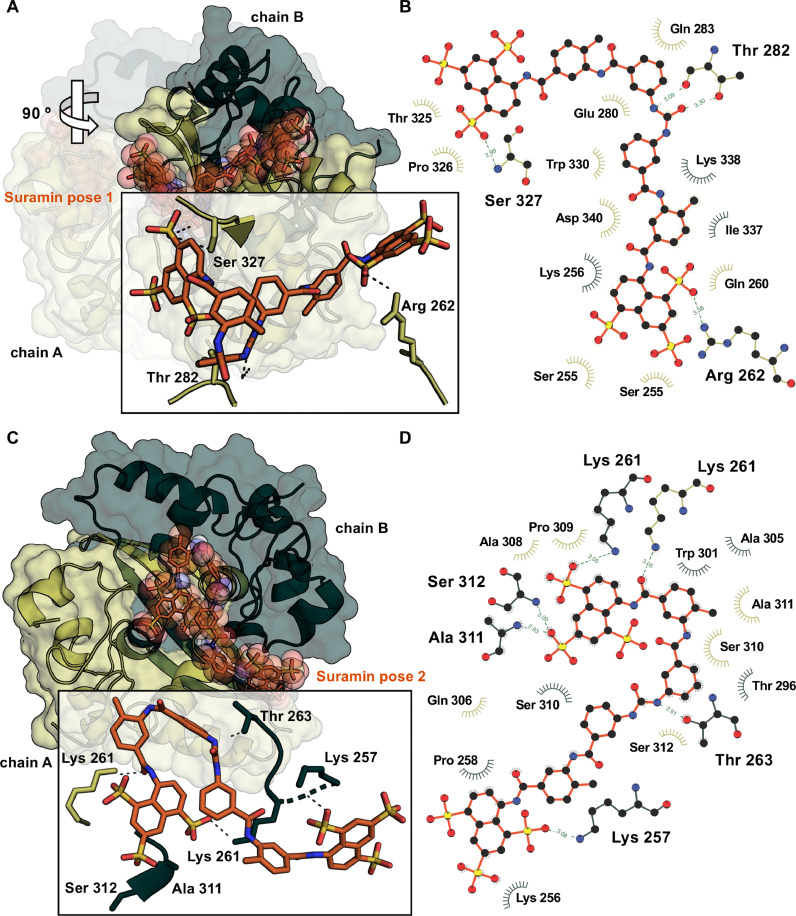
Model and interaction map for Suramin binding to SARS-CoV-2 N CTD. Cartoon 3D representation of Suramin putative binding mode to SARS-CoV-2 N CTD dimer interface, according to best pose by molecular docking for binding site 1. (B) 2D-diagram of predicted Suramin-residue interactions within the N CTD dimer interface. (C) Cartoon 3D representation of Suramin putative binding mode to SARS-CoV-2 N CTD dimer RNA binding groove, according to best pose by molecular docking for binding site 2. (D) 2D-diagram of predicted Suramin-residue interactions within the N CTD dimer RNA binding groove. 3D-intermolecular interactions at binding sites are zoomed in the inset; H-bonds and hydrophobic contacts are shown as dashed lines with distances between atoms indicated in angstrom and as arcs with spokes, respectively.

Therefore, as next step, we computationally investigated the Suramin mode of binding to the globular domains of SARS-CoV-2 N by blind molecular docking simulations. Analysis of Suramin docking to SARS-CoV-2 N NTD showed that the compound is predicted to accommodate along the cleft made up by the protruding basic finger and the upper part of the palm in the right hand-shaped (β-hairpin) - (loop I) - (β-sheet core) - (loop II) fold of the protein domain (Kang et al., 2020; Peng et al., 2020) (Fig. 3A and Supplementary Figure S1). Noteworthy, this cleft overlaps with the positively charged region where Suramin analogs were also predicted to dock (Dey et al., 2021), and neighbors the NTD binding pockets of potential ligands previously identified by virtual screening of synthetic small molecules (Sarma et al., 2021; Yadav et al., 2021; Hu et al., 2021) and phytocompounds (Rolta et al., 2021) libraries. In the best pose of our model Suramin is predicted to interact with residues Ala55 and Arg107, which are part of the NTD 5′- monophosphate ribonucleotide binding site (Kang et al., 2020; Dang et al., 2021), as well as with amino acids Arg92, Arg93 and Ile94, which were also independently predicted as docked by Suramin in another study published during the preparation of this manuscript (Guo et al., 2023) and were found by mutagenesis to be crucial for the NTD RNA binding function due to their interaction with the nucleic acid backbone (Dinesh et al., 2020) (Fig. 3B and Supplementary Fig. S2). Analysis of Suramin docking to SARS-CoV-2 N CTD revealed two binding sites, one corresponding to a cavity located laterally at the interface where the β-hairpins of the two CTD monomers associate into a dimeric rectangular slab (Zinzula et al., 2021; Peng et al., 2020) (Fig. 4A and Supplementary figure S1), and the other one lying along the basic groove predicted to accommodate the viral genome RNA backbone, made up by the 55a-helices and loops that form the concave face of the CTD dimer (Zinzula et al., 2021; Chauhan et al., 2022) (Fig. 4C and Supplementary figure S1). Notably, not only does the location of these sites resemble that of the binding pockets identified in a previous virtual screening study and targeted by small-molecule ligands (Chauhan et al., 2021), but their prediction is also in agreement with the presence of two inflection points in the thermal curve of the CTD (Zinzula et al., 2021), as well as with our herein shown experimental observation of the shift undergone by them during Nano-DSF in the presence of Suramin. The two best poses of our model show that in the first binding site Suramin is predicted to interact with residues Arg262, Thr282 and Ser327 (Fig. 4B), whereas in the second binding site Suramin is predicted to establish interactions with amino acids Lys257, Lys261, Thr263, Ala311 and Ser312 (Fig. 4D). Arg262 is known to be involved in the CTD binding to ATP purine rings and to modulate the RNP complex formation with viral genomic RNA (Dang et al., 2022), whereas Ser312 and Ser327 are known to be involved in critical interactions for the CTD dimerization and multimerization, respectively (Zhou et al., 2020b; Yang et al., 2021). Furthermore, Lys257, Lys261, Thr263 and Arg262 are all components of the residue cluster that form the basic groove where binding of viral genome putatively occurs (Peng et al., 2020; Zinzula et al., 2021; Mercaldi et al., 2022) and that were also found to be targets of post-translational modifications, putatively for the modulation of RNA binding activity (Stukalov et al., 2021) (Fig. 4D and Supplementary Fig. S3). Therefore, results from molecular docking simulations suggest that interaction of Suramin with SARS-CoV-2 N NTD and CTD might interfere with the RNA binding function of the two domains. Notably, this is in line with previous findings showing that, given its highly negative charge, Suramin tends to interact with basic residue patches on regions deputed to bind nucleic acids, such as those present in viral nucleoproteins and polymerases (Mastrangelo et al., 2012; Jiao et al., 2013; Ellenbecker et al., 2014; Yin et al., 2021; Yuan et al., 2022).

### 3.3 Suramin prevents ssRNA binding by SARS-COV-2 N NTD and CTD *in vitro*

**Fig. 5:**
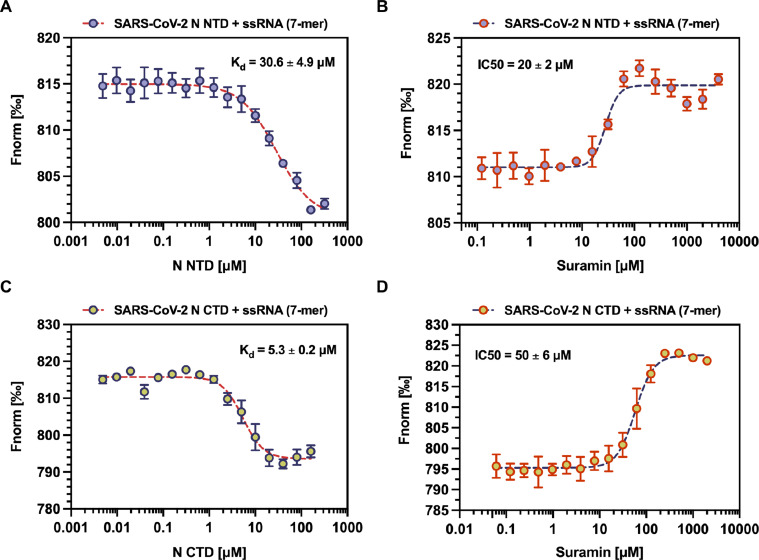
Suramin inhibition of SARS-CoV-2 N NTD and CTD ssRNA binding. (A) MST analysis of SARS-CoV-2 N NTD binding and affinity to Cy5-ssRNA 7-mer showing low-micromolar apparent K_d_ (30.6 ± 4.9 μM). (B) MST analysis of SARS-CoV-2 N NTD interaction with Cy5-ssRNA 7-mer showing Suramin dose-dependent inhibition of ssRNA binding with low-micromolar IC_50_ (≈ 20 ± 2 μM). (C) MST analysis of SARS-CoV-2 N CTD binding and affinity to Cy5-ssRNA 7-mer showing low-micromolar apparent K_d_ (5.3 ± 0.3 μM). (D) MST analysis of SARS-CoV-2 N NTD interaction with Cy5-ssRNA 7-mer showing Suramin dose-dependent inhibition of ssRNA binding with low-micromolar IC_50_ (≈ 50 ± 6 μM). Dose-response curves were obtained by non-linear regression fitting (deep-koamaru blue and pomegranate orange dashed lines) on experimental points (blue bell and wild willow green circles). Experimental points, K_d_ and IC_50_ values are represented as averages and standard deviations (deep-koamaru blue and pomegranate orange bars) from at least three independent experiments.

A general assumption is that coronaviral N proteins encapsidate the viral genome by interacting with ssRNA in a sequence-independent fashion (Chang et al., 2014; Zhang et al., 2022), whereas either the full-length SARS-CoV-2 N protein or its isolated NTD and CTD truncations have been experimentally found capable of binding, with different affinities, to a variety of nucleic acid moieties such as ssRNA (Guo et al., 2023; Zinzula et al., 2021; Dinesh et al., 2020; Kang et al., 2020; Zhou et al., 2020b; Yang et al., 2021; Ye et al., 2021; Jack et al., 2021; Lu et al., 2021; Perdikari et al., 2020; Carlson et al., 2020), ssDNA (Zhou et al., 2020b; Zhao et al., 2021), double-stranded DNA (dsDNA) (Zeng et al., 2020; Zhou et al., 2020b) and stem-loop dsRNA oligomers (Dinesh et al., 2020; Dang et al., 2022; Mercaldi et al., 2022; Ye et al., 2021; Jack et al., 2021). Moreover, given that it was postulated that the building blocks of the coronaviral RNP complex accommodate each a ssRNA of at least 7 bp in length (Chang et al., 2014), we previously adopted this nucleic acid - in the form of a Cy5-fluorolabeled synthetic oligomer whose sequence corresponds to the beginning of the SARS-CoV-2 genome - as the biologically relevant substrate to mimic the ssRNA tract enwrapped by a single SARS-CoV-2 N protomer (Zinzula et al., 2021). Hence, we used MST, a technique that allows to infer protein-nucleic acid interactions from the differential migration through a temperature gradient of a given bimolecular complex with respect to its isolated components (Jerabek-Willemsen et al., 2011), to quantitatively characterize the ssRNA binding properties of SARS-CoV-2 N NTD and CTD towards the Cy5-ssRNA heptameric probe. As shown, the SARS-CoV-2 N NTD bound this oligomer with micromolar affinity, displaying an apparent K_d_ value of 30.6 ± 4.9 μM (Fig. 5A, Supplementary Table 2 and Supplementary figure S4), which is consistent with affinity values reported for NTD binding to similar ssRNA substrates (Guo et al., 2023; Dinesh et al., 2020; Kang et al., 2020). Similarly, the SARS-CoV-2 N CTD bound the Cy5-ssRNA 7-mer with a K_d_ value of 5.3 ± 0.2 μM (Fig. 5C, Supplementary Table 2 and Supplementary figure S4), which agrees with our previous observation (Zinzula et al., 2021) and in the same order of magnitude of previously reported affinity values for CTD binding to ssRNA of similar length (Yang et al., 2021). Furthermore, to assess whether Suramin would affect SARS-CoV-2 N NTD and CTD ssRNA binding, we monitored the change in the thermophoresis profile undergone by the two protein-RNA complexes in the presence of increasing concentrations of compound. When titrated against a fixed amount of protein and substrate, Suramin inhibited SARS-CoV-2 N NTD binding of the Cy5-ssRNA 7-mer in a dose-dependent manner, yielding a IC_50_ value of 20 ± 2 μM (Fig. 5B, Supplementary Table 2 and Supplementary figure S4). Similarly, binding of SARS-CoV-2 N CTD to the heptameric ssRNA probe was inhibited by Suramin in a dose-dependent manner and with an IC_50_ value of 50 ± 6 μM (Fig. 5D, Supplementary Table 2 and Supplementary figure S4). Noteworthy, in both cases the IC_50_ value obtained during the MST titration experiments is consistent with that of the apparent K_d_ value measured by the Nano-DSF ones, supporting the notion of a direct correlation between the inhibitory effect exerted by Suramin towards the RNA binding function and the affinity of the compound for each protein domain. Altogether, results indicate that interaction of Suramin is sufficient to prevent the SARS-CoV-2 N NTD and CTD ability to form a complex with ssRNA.

### 3.4 Suramin inhibits SARS-COV-2 N genome packaging function *in vitro*

**Fig. 6:**
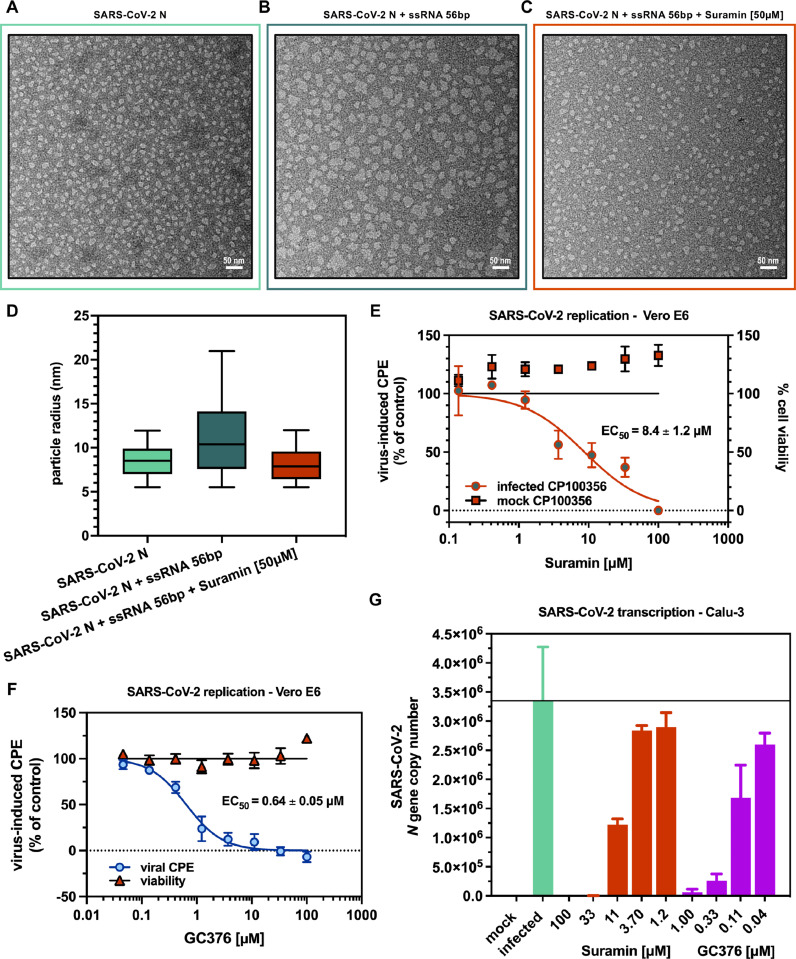
Suramin inhibition of SARS-CoV2 N genome packaging and virus replication. Representative micrographs from negative stain EM showing: (A) purified SARS-CoV-2 N in the absence of ssRNA; (B) *in vitro* reconstituted SARS-CoV-2 RNP complex-like intermediates formed upon incubation of SARS-CoV-2 N with ssRNA 56-mer; (C) inhibition of SARS-CoV-2 RNP complex-like intermediates formation in the presence of 50 μM Suramin. (D) Analysis of particle radius distribution between negative stain EM micrograph datasets of purified SARS-CoV-2 N in the absence of ssRNA (left box, lime green), RNP complex-like intermediates formed upon SARS-CoV-2 N interaction with ssRNA 56-mer in the absence (middle box, deep teal) or in the presence (right box, pomegranate orange) of 50 μM Suramin; median is shown as continuous line in the middle of each box, with top and bottom representing the 75th and 25th percentiles, respectively (**** *P* < 0.0001, unpaired two-tailed Student's *t*-test, *N* = 2). (E) Effect of Suramin on SARS-CoV-2 replication in Vero E6-GFP cells. (F) Effect of GC376 on SARS-CoV-2 replication in Vero E6-GFP cells. Results were expressed as percentage of virus-induced CPE over uninfected and untreated control (circles, deep teal/pomegranate orange). Suramin cytotoxicity was tested in parallel, in uninfected wells on the same plate; results were expressed as percentage of cellular viability over untreated control (squares, pomegranate orange/black). EC_50_ was calculated with non-linear regression function of two independent experiments on triplicate. (G) Effect of Suramin and GC376 on SARS-CoV-2 replication in Calu-3 cells. Results were expressed as SARS-CoV-2 *N* gene copy number, EC_50_ was calculated with non-linear regression function of the percentage of viral gene copy number over the infected untreated sample; data are the results of two independent experiments in duplicate. Statistical significance was evaluated by one-way analysis of variance (ANOVA, *N* = 3) followed by post-hoc Dunnett test (Supplementary Table 3).

Structural studies revealed that SARS-CoV-2 N enwraps ssRNA as a dimeric protomer (Zeng et al., 2020; Zinzula et al., 2021; Ribeiro-Filho et al., 2022), and that N dimers assemble onto viral genome as beads-on-a-string to form a shell-like, barrel-shaped RNP complex (Yao et al., 2020; Klein et al., 2020; Carlson et al., 2022). Moreover, several works provided evidence that SARS-CoV-2 N undergoes liquid-liquid phase separation (LLPS) with RNA, during which multivalent protein-protein and protein-RNA interactions involving both the NTD and the CTD, as well as the three IDRs, drive a dynamic transition that, from liquid-like spherical condensates through solid-like and amorphous ones, bring to the formation of an RNP complex that ensures full-genome compaction (Jack et al., 2021; Lu et al., 2021; Perdikari et al., 2020; Carlson et al., 2020; Savastano et al., 2020; Iserman et al., 2020; Chen et al., 2020a; Wang et al., 2021; Cubuk et al., 2021). Furthermore, small molecules were found able to disrupt such condensates, laying the conceptual foundations for the targeting of SARS-CoV-2 genome packaging as a strategy to develop Covid-19 therapeutics (Jack et al., 2021; Iserman et al., 2020). Hence, with this is mind, we wanted to assess whether interaction of Suramin with SARS-CoV-2 N NTD and CTD, and its inhibition of their ssRNA binding activity, would also impair the formation of N-RNA condensates *in vitro*. To this aim, we incubated the recombinant full-length SARS-CoV-2 N with a synthetic ssRNA oligomer whose sequence corresponds to the first 56 bases of the SARS-CoV-2 genome, and comparatively analyzed by negative stain EM the reconstitution of RNP complex-like intermediates in the absence and in the presence of Suramin. In agreement with previous observations (Carlson et al., 2020; Ribeiro-Filho et al., 2022, 2022), the purified recombinant SARS-CoV-2 N sample displayed a heterogeneous population of round-shaped particles, with the most represented ones having a donut-like architecture (Fig. 6A) and an average radius of ∼ 8.5 nm (Fig. 6D). When mixed to the 56 bp-long ssRNA, SARS-CoV-2 N formed instead large and amorphous, solid-like aggregates (Fig. 6B) with an average radius of ∼ 11.2 nm (*P* < 0.0001) (Fig. 6D and Supplementary Table 1), which also resemble in size and shape previously reported N-RNA condensates that were seen forming in the presence of nucleic acids with similar features (Jack et al., 2021; Lu et al., 2021; Ribeiro-Filho et al., 2022). By contrast, concomitant addition to the N-RNA mixture of Suramin at 50 μM final concentration (*i.e.*, close to the highest IC_50_ value obtained in our MST RNA-binding inhibition curves) fully inhibited the formation of N-RNA condensates (Fig. 6C), reverting the average radius of SARS-CoV-2 N particles to ∼ 8.1 nm (*P* < 0.0001), a value comparable to the one shown by the protein sample in the absence of ssRNA (Fig. 6D and Supplementary Table 1). Therefore, paralleling those obtained by Nano-DSF and MST, and in line with predictions from molecular docking simulations, the results from the EM analysis indicate that, upon interaction with the NTD and the CTD and inhibition of their ssRNA binding function, Suramin hampers the reconstitution of RNP complex-like intermediates *in vitro*, thereby interfering with the packaging of viral genome by SARS-CoV-2 N.

### 3.5 Suramin inhibits SARS-CoV-2 replication *in cellulo*

Recent structural works showed that Suramin interacts with NoV (Mastrangelo et al., 2012), SARS-CoV-2 (Yin et al., 2021) and EBOV (Yuan et al., 2022) RdRp complexes and inhibits their enzymatic activity, which led to the notion of Suramin as inhibitor of viral polymerases and therefore as suitable model molecule for antiviral studies aimed at targeting the viral RNA synthesis process. Moreover, notwithstanding its affinity towards positively charged surfaces on proteins involved in RNA processing (Dey et al., 2021), it is worth noting that Suramin was reported to fail in interfering with the oligomerization of the RNA-helicase domain of the EBOV RdRp co-factor VP35 (Zinzula et al., 2022), suggesting that, rather than being promiscuous and generalist, the inhibitory role of this molecule is instead performed under specific interaction constrains. In this regard, the fact that Suramin also prevents ssRNA encapsidation by SARS-CoV-2 N would expand the framework to another fundamental step of the coronaviral life cycle, where this compound can be adopted as tool for undertaking cell-based mechanistic studies to explore SARS-CoV-2 N antiviral target potential. Hence, to validate our biophysical and biochemical data by assessing the Suramin inhibitory effect in a cellular context, we tested Suramin against the CPE induced by SARS-CoV-2 replication in a Vero E6 cells. To this aim we employed a Vero E6 cell line that constitutively express the GFP under the control of cytomegalovirus (CMV) promoter, and tested SARS-CoV-2 replication in presence of CP100356, a nontoxic inhibitor of the efflux transporter P-glycoprotein, which is highly expressed in Vero E6, to not underestimate the effect of the compound due to its depletion from the cell. As positive control for the inhibition, the protease inhibitor GC376 was used. Results confirmed that Suramin inhibited SARS-CoV-2 replication in Vero E6-GFP cells with a EC_50_ value of 8.4 ± 1.2 μM (Fig. 6E and Supplementary Table 2), whereas GC376 showed an EC_50_ value of 0.64 ± 0.05 μM (Fig. 6F and Supplementary Table 2), with no sign of cytotoxicity at the highest concentration tested (100 μM), which agrees with previously reported data in other cell lines (Salgado-Benvindo et al., 2020; Yin et al., 2021). Furthermore, as orthogonal confirmation of viral load reduction, a replication assay coupled to RT-qPCR for the detection of SARS-CoV-2 *N* gene as viral biomarker was performed on Calu-3 cells, a human lung adenocarcinoma epithelial cell line that resembles the airway epithelium, is more biologically relevant to SARS-CoV-2 infection and better recapitulates its pathogenesis, and that - because of its low expression levels of efflux pumps - mitigates artifacts on compound activity due to drug extrusion. As shown, both compounds inhibited SARS-CoV-2 *N* gene transcription in Calu-3 cells, showing an EC_50_ value of 8.16 ± 1.4 μM and of 0.068 ± 0.0032 μM for Suramin and GC376, respectively (Fig. 6G and Supplementary Tables 2 and 3), whereas neither Suramin, nor GC376, had any effect on the transcription level of GAPDH control gene at the concentrations tested (Supplementary figure S5). Therefore, altogether results from *in cellulo* assays show that Suramin inhibits SARS-CoV-2 replication, noteworthy displaying similar potency in two different cell lines, one of which is human lung-derived.

### 3.6 Suramin reduces SARS-COV-2 N IFN-I antagonism *in cellulo*

**Fig. 7:**
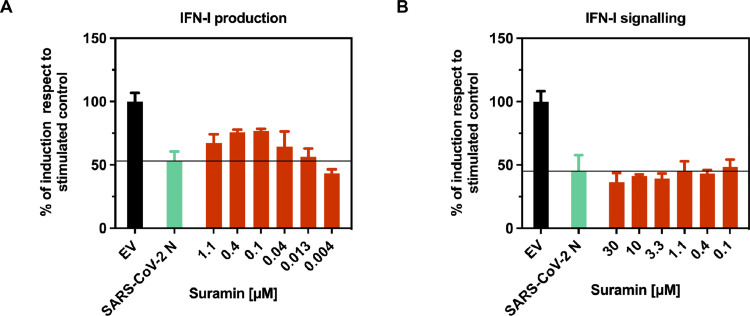
Suramin inhibition of SARS-CoV-2 N IFN-I antagonism. (A) Effect of Suramin on SARS-CoV-2 N inhibition of IFN-I promoter activation upon stimulation with viral RNA. (B) Effect of Suramin on SARS-CoV-2 N inhibition of ISRE promoter activation upon stimulation with IFN-α. Results were expressed as percentage of induction between Suramin untreated control (lime green bar) and Suramin treated samples (pomegranate orange bars) over unstimulated control transfected with the empty vector (black bar), and plotted as mean plus standard deviation of two independent experiments on triplicate (EV, empty vector). Statistical significance was evaluated by one-way analysis of variance (ANOVA, *N* = 3) followed by post-hoc Dunnett test (Supplementary Table 3).

In addition to the primary function of preserving genomic content and providing a scaffold for viral RNA replication and transcription, to the SARS-CoV-2 N protein is also ascribed the ability of counteracting the host innate immune antiviral response based on the IFN-I production (Li et al., 2020; Liu et al., 2021). Particularly, SARS-CoV-2 N was reported to downregulate, by hitting at several levels the signaling cascade triggered by retinoic acid-inducible gene I (RIG-I)-like receptors (RLRs), the induction of IFN- α/β and IFN-λ (Chen et al., 2020b; Oh and Shi, 2021; Gori Savellini et al., 2021). Moreover, the sole CTD was found itself indispensable for the exertion of the IFN-I antagonism properties of the protein (Wu et al., 2021). In addition, SARS-CoV-2 N was found capable of antagonizing the IFN-I signaling by interacting with Janus kinase (Jak) signal transducer and activator of transcription (STAT) 1 and STAT2, thereby inhibiting their phosphorylation and nuclear translocation. Also, in this case both the NTD and CTD, but not the IDR_3_, were required for the exertion of such inhibitory function (Mu et al., 2020). Hence, we sought to assess whether the interaction of Suramin would also affect the inhibitory capabilities of SARS-CoV-2 N towards the host cell IFN-I response, and to this aim we tested Suramin in a luciferase-based reporter assay established to evaluate the effect of SARS-CoV-2 N on either the IFN-I or the ISRE promoter activation. When tested towards the inhibition by SARS-CoV-2 N of the IFN-I promoter activation upon cell stimulation with transfected viral RNA, Suramin was able to significantly revert the IFN-I promoter activation and to suppress the SARS-CoV-2 N IFN-I antagonism already from 0.1 μM concentration, however with an overall effect that did not exceed 50 % of reversion. (Fig. 7A and Supplementary Table 3). Instead, when tested towards the inhibition of ISRE promoter activation by SARS-CoV-2 N upon cell stimulation with IFN-α, Suramin showed no significant effect in the range of concentrations tested (Fig. 7B and Supplementary Table 3). In line with our biophysical data and *in silico* predictions, this discrepancy could have an explanation in the fact that Suramin interaction with the RNA binding pocket of NTD and CTD may affect the N protein capability to *hide* or *mask* the RNA from RLRs recognition, whereas the same interaction could be ineffective in preventing the *hit* of STAT1 and STAT2 via direct interaction (Zinzula and Tramontano, 2013). Nevertheless, altogether these results suggest that, in addition of being capable of inhibiting SARS-CoV-2 replication by interfering with the viral genome encapsidation step, Suramin can also abolish, at least in part, the SARS-CoV-2 N IFN-I antagonism, however limiting its counteraction towards the inhibition of IFN-I production and with no significant effects on that of the IFN-I signaling pathway.

## 4 Conclusions

In the present study we demonstrate that the century-old drug Suramin, whose multifaceted therapeutic potential has been explored over decades against several pharmacological targets (Wiedemar et al., 2020), interacts with both NTD and CTD of the SARS-CoV-2 N protein, inhibiting their ssRNA binding function. *In silico* models illustrate the putative structural basis for such inhibition, in which Suramin positioning along the NTD basic cleft and the CTD dimer basic groove hampers the establishment of electrostatic interactions that would otherwise allow accommodation of a nucleic acid backbone. Such predictions correlate with the evidence that Suramin abolishes the formation *in vitro* of N-ssRNA condensates, necessary intermediates for genome encapsidation by SARS-CoV-2 N into a functional RNP-complex. Furthermore, at the same clinically relevant concentration range at which it hampers N-ssRNA binding, Suramin inhibits SARS-CoV-2 replication and reverts the IFN-I antagonism of SARS-CoV-2 N. Noteworthy, albeit with slight differences in affinities due to diverse methodology, biochemical environment and type of RNA substrate, our findings are corroborated by similar results recently published during the preparation of our manuscript, in which Suramin was found to interact with SARS-CoV-2 N NTD (Guo et al., 2023) and where it emerged as positive hit during a high throughput screening campaign targeting full-length SARS-CoV-2 N (Mercaldi et al., 2022). Also, in line with previous works that reported Suramin inhibition of nucleoprotein-RNA interactions in severe fever with thrombocytopenia syndrome virus (Jiao et al., 2013) and Rift Valley fever virus (Ellenbecker et al., 2014), our findings expand to SARS-CoV-2 the potential benefit of using this molecule as antiviral to target the genome packaging and nucleocapsid assembly steps during infections by RNA viruses. However, because of the well-known side effects (Wiedemar et al., 2020) and the intrinsic limitations in the conclusions drawable from *in vitro* studies, results herein shown should not be overinterpreted for the direct repurposing of Suramin as therapeutic choice to treat COVID-19 patients. Rather, we emphasize that the main value of exploiting the inhibitory properties of Suramin towards SARS-CoV-2 N-RNA interaction resides in providing a proof of concept to encourage further medicinal chemistry efforts, which eventually could result in the design of a new generation of less cytotoxic and more effective analogs and derivatives.

## CRediT authorship contribution statement

**L.Z.**: conceived the study. **I.N. and L.Z.**: designed and performed molecular cloning. **I.B. A.C., J.B., C.B., J.M. and L.Z.**: carried out all biophysical, biochemical, computational and biological experiments. **I.B., A.C., I.N. and L.Z.**: performed data analysis and interpretation. **E.T. and L.Z.**: supervised experimental design and data interpretation. **L.Z. and A.C.**: drafted the manuscript. All authors contributed to experimental design, data analyses and manuscript review.

## Declaration of Competing Interest

The authors declare that they have no known competing financial interests or personal relationships that could have appeared to influence the work reported in this paper:

## Data Availability

Data will be made available on request.

## Appendix Supplementary materials

Image, application 1
