# Supplementary material for: Suramin inhibits SARS-CoV-2 nucleocapsid phosphoprotein genome packaging function
Source: Virus Res. 2023 Sep 15;336:199221. doi: 10.1016/j.virusres.2023.199221 (PMC10514558; doi:10.1016/j.virusres.2023.199221)
Supplement: Supplementary file 1 [file mmc1.pdf]

Suramin inhibits SARS-CoV-2 nucleocapsid phosphoprotein genome packaging function

Irene Boniardi [a,1], Angela Corona [b,1], Jerome Basquin [c], Claire Basquin [c],
Jessica Milia [b], István Nagy [a,d], Enzo Tramontano [b,*] Luca Zinzula [a,*,2]

[a] Max Planck Institute of Biochemistry, Department of Molecular Structural Biology, 82152 Martinsried, Germany
[b] University of Cagliari, Department of Life and Environmental Sciences, 09042 Monserrato, Italy
[c] Max-Planck Institute of Biochemistry, Department of Structural Cell Biology, 82152 Martinsried, Germany
[d] Eszterházy Károly Catholic University, Center of Research and Development, 3300 Eger, Hungary
*Corresponding authors: zinzula@biochem.mpg.de ; tramon@unica.it

[1]These authors made equal contribution to this work
[2]Current address: iHuman Institute ShanghaiTech University, 201210, Shanghai, China; zinzulal@shanghaitech.edu.cn

Supplemental Information

Supplementary Table 1.

Related to figures 1C, 1D, 2A, 2B, 2C, 2D: Suramin effect on SARS-CoV-2 N, N-NTD and N-CTD Tm				
**SARS-CoV-2 N**	**full length (Tm_1)**	**NTD**	**CTD (Tm_1)**	**CTD (Tm_2)**
control	45.9 ± 0.1 ºC	53.2 ± 0.1 ºC	49.1 ± 0.1 ºC	53.7 ± 0.1 ºC
+ Suramin (100 μM)	42.0 ± 0.1 ºC	42.0 ± 0.1 ºC	42.0 ± 0.1 ºC	51.7 ± 0.1 ºC
Unpaired two-tailed Student's t-test				
P value	<0.0001	<0.0001	<0.0001	<0.0001
P value summary	****	****	****	****

Related to figures 6D: Suramin effect on SARS-CoV-2 RNP complex-like intermediates formation		
**SARS-CoV-2 N**	**+ ssRNA 56-mer**	**+ ssRNA 56-mer, + Suramin (50 μM)**
vs.	*vs.*	*vs.*
Unpaired two-tailed Student's t-test		
P value	<0.0001	<0.0001
P value summary	****	****

Supplementary Table 1, related to figures 1, 2 and 6. Statistical analysis of *in vitro* experiments Analysis of the statistical significance of the shift in Tm of SARS-CoV-2 N, N-NTD and N-CTD in the presence of 100 μM Suramin, and of the change in particle radius on negative stain EM of SARS-CoV-2 N in the presence of ssRNA 56-mer, with or without 50 μM Suramin, respectively. The number of asterisks proportionally flags the level of statistical significance (****, highly significant).

Supplementary Table 2.

Related to figures 2E, 2F, 5A, 5B, 5C, 5D, 6E, 6F, 6G: summary of K_d, IC_{50} and EC_{50} values				
**Suramin**	**NTD**	**CTD $(K_d)_1$**	**CTD $(K_d)_2$**	
K_d *vs.* SARS-CoV-2 N	29.5 ± 0.7 μM	31.4 ± 1.0 μM	248.2 ± 10.0 μM	
**Cy5-ssRNA 7-mer**	**NTD**	**CTD**		
K_d *vs.* SARS-CoV-2 N	30.6 ± 4.9 μM	5.3 ± 0.2 μM		
**Suramin**	**NTD**	**CTD**		
IC_{50} *vs.* SARS-CoV-2 N + Cy5-ssRNA 7-mer	20.0 ± 2.0 μM	50.0 ± 6.0 μM		
**Suramin**	**Vero E6**	**Calu-3**		
EC_{50} *vs.* SARS-CoV-2 replication	8.4 ± 1.2 μM	8.2 ± 1.4 μM		
**GC376**	**Vero E6**	**Calu-3**		
EC_{50} *vs.* SARS-CoV-2 replication	0.64 ± 0.05 μM	0.07 ± 0.03 μM		

Supplementary Table 2, related to figures 2, 5 and 6. Summary of K_d, IC_{50} and EC_{50} values Low-micromolar affinity of Suramin and Cy5-ssRNA 7-mer for SARS-CoV-2 N NTD and CTD; inhibitory effect of Suramin on ssRNA binding by SARS-CoV-2 N NTD and CTD; inhibitory effect of Suramin and control compound GC376 on SARS-CoV-2 replication in Vero and Calu-2 cells

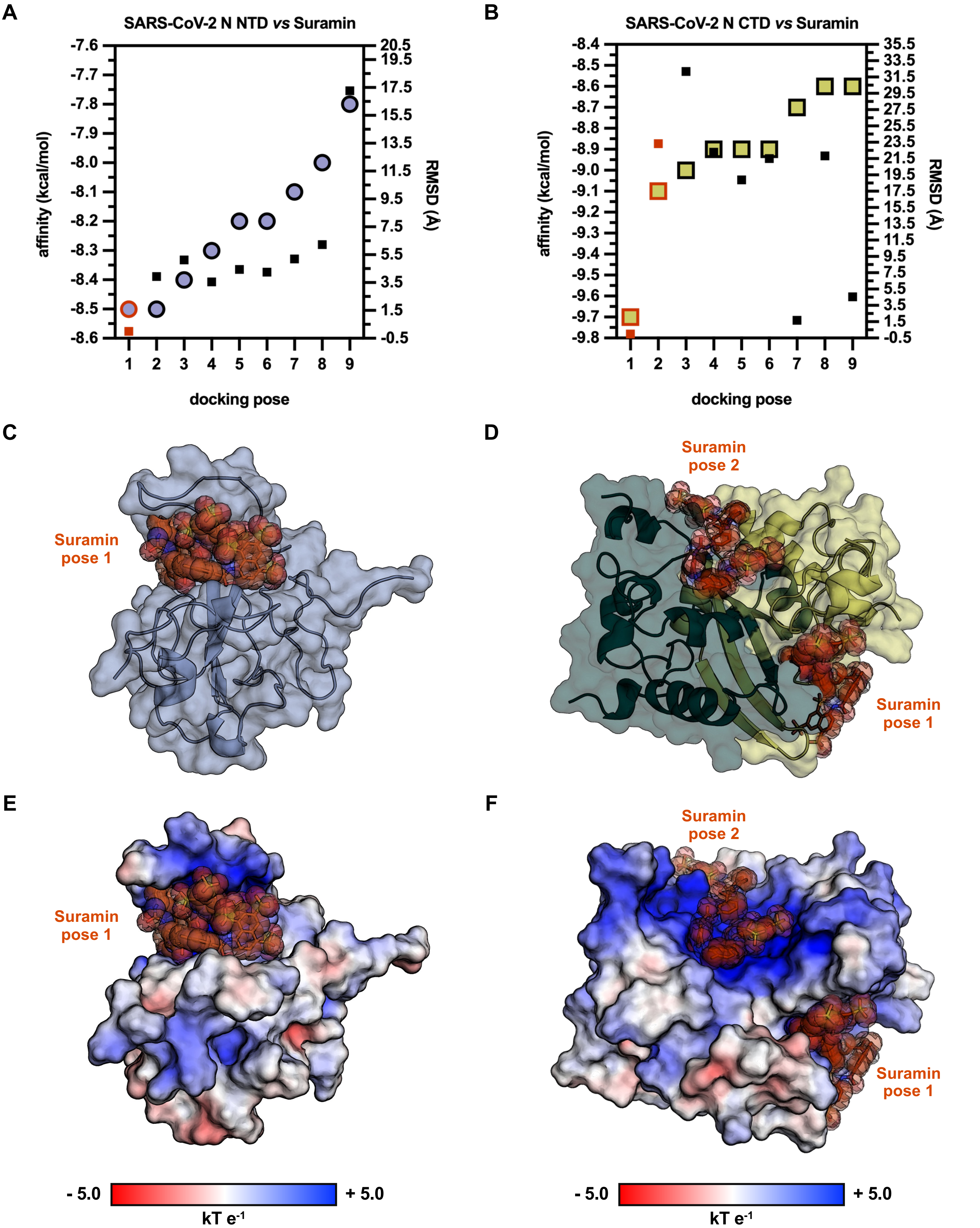

Supplementary figure S1, related to figures 3 and 4. Predicted binding modes of Suramin to SARS-CoV-2 N NTD and CTD (A) Molecular docking poses of Suramin bound to SARS-CoV-2 N NTD ranked by their affinity and root mean squared deviation (RMSD). **(B)** Molecular docking poses of Suramin bound to SARS-CoV-2 N CTD ranked by their affinity and root mean squared deviation (RMSD). Best poses for each unique binding site are highlighted in pomegranate orange. **(C)** Cartoon 3D representation of the molecular docking best pose for Suramin binding to SARS-CoV-2 N NTD. **(D)** Cartoon 3D representation of the molecular docking two best poses (one for each unique binding site) for Suramin binding to SARS-CoV-2 N dimeric CTD. **(E)** Electrostatic surface potential analysis of SARS-CoV-2 N NTD in complex with one Suramin molecule; red, white and blue regions represent areas with negative, neutral and positive electrostatic potential, respectively (scale from -5.0 to +5.0 kT e^{-1}). **(F)** Electrostatic surface potential analysis of SARS-CoV-2 N dimeric CTD in complex with two Suramin molecules; red, white and blue regions represent areas with negative, neutral and positive electrostatic potential, respectively (scale from -5.0 to +5.0 kT e^{-1}).

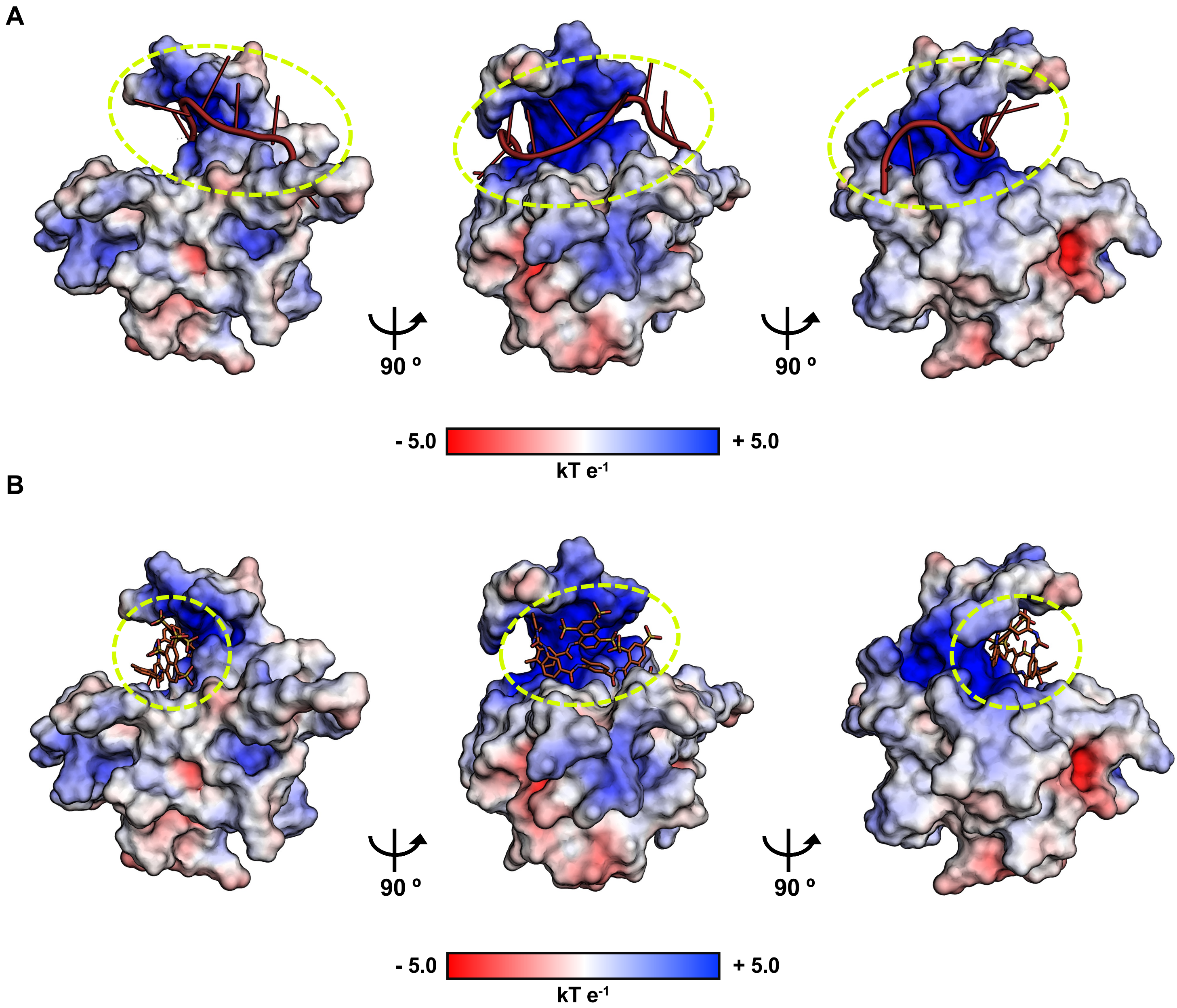

Supplementary figure S2, related to figure 3. Suramin binding to SARS-CoV-2 N NTD ssRNA binding site (A) Electrostatic surface potential analysis of SARS-CoV-2 N NTD in complex with ssRNA 10-mer (PDB: 7ACT) shown in three orientations. **(B)** Electrostatic surface potential analysis of SARS-CoV-2 N NTD in complex with Suramin (this work, molecular docking from PDB: 7ACT), shown in three orientations. The overlapping binding site between the nucleic acid and the compound within the basic cleft of SARS-CoV-2 N NTD is highlighted as dashed circle in electric lime. Red, white and blue regions in the two panels represent areas with negative, neutral and positive electrostatic potential, respectively (scale from -5.0 to +5.0 kT e^{-1}).

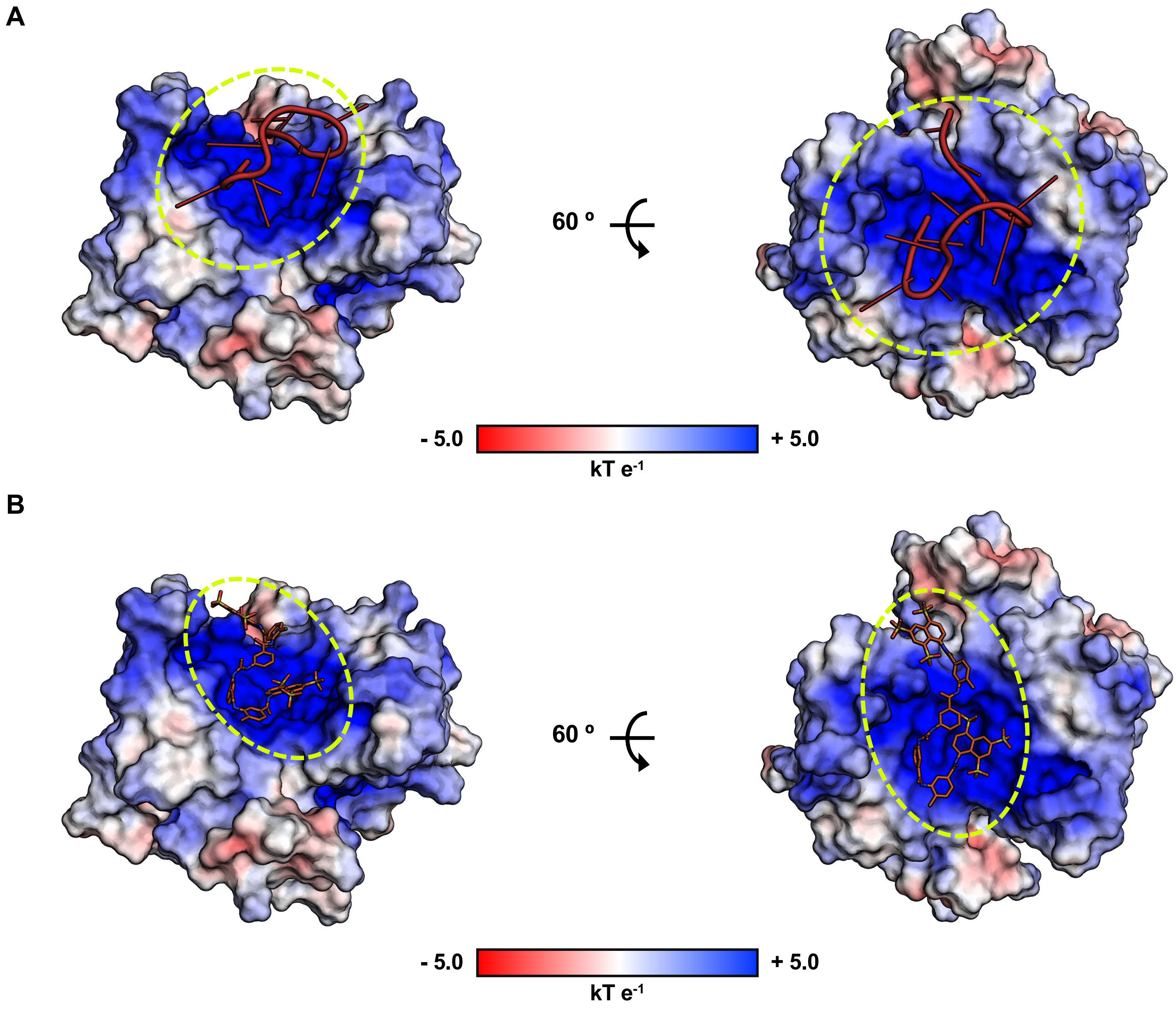

Supplementary figure S3, related to figure 4. Suramin binding to SARS-CoV-2 N dimeric CTD putative ssRNA binding site (A) Electrostatic surface potential analysis of SARS-CoV-2 N dimeric CTD in complex with ssRNA 10-mer (this work, molecular docking from PDB: 6YUN and PDB:7ACT) shown in two orientations. **(B)** Electrostatic surface potential analysis of SARS-CoV-2 N dimeric CTD in complex Suramin (this work, molecular docking from PDB: 6YUN, binding site 2), shown in two orientations. The overlapping binding site between the nucleic acid and the compound within the basic groove of SARS-CoV-2 N dimeric CTD is highlighted as dashed circle in electric lime. Red, white and blue regions in the two panels represent areas with negative, neutral and positive electrostatic potential, respectively (scale from -5.0 to +5.0 kT e $^{-1}$).

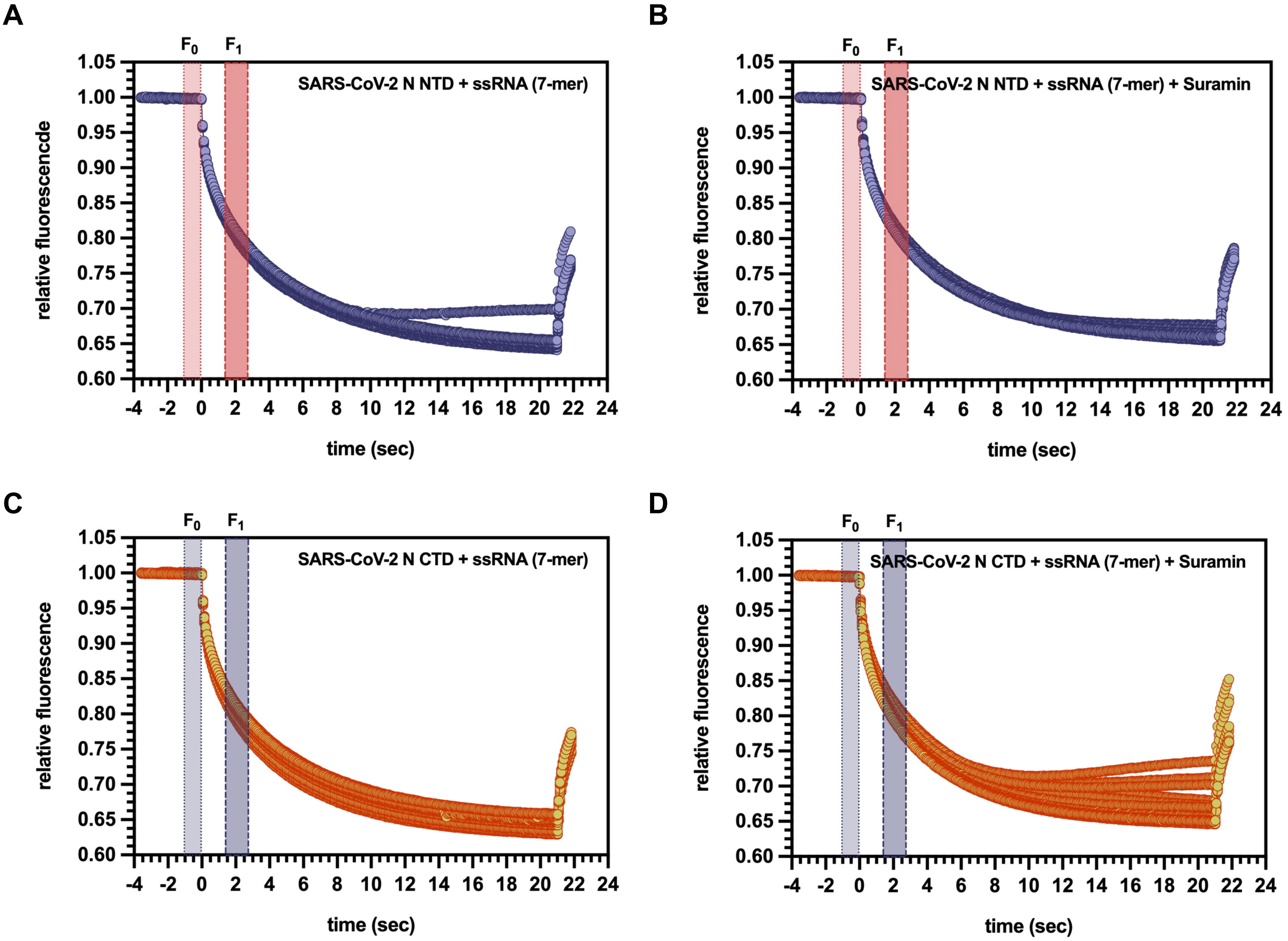

Supplementary figure S4, related to figure 5. SARS-CoV-2 N NTD and CTD binding to ssRNA 7-mer in the presence or absence of Suramin **(A)** MST traces for the titration of SARS-CoV-2 N NTD against Cy5-ssRNA 7-mer. **(B)** MST traces for the titration of Suramin against SARS-CoV-2 N NTD and Cy5-ssRNA 7-mer. **(C)** MST traces for the titration of SARS-CoV-2 N dimeric CTD against Cy5-ssRNA 7-mer. **(D)** MST traces for the titration of Suramin against SARS-CoV-2 N dimeric CTD and Cy5-ssRNA 7-mer. Cold (F_0) and heated (F_1) state regions for the measurement of the Fnorm ($F_1 F_0^{-1}$ ‰) ratio are indicated as dotted and dashed rectangular areas, respectively.

Supplementary Table 3.

Related to figure 6G: inhibition of SARS-CoV-2 *N* gene transcription						
**Suramin**						
[μM]	100	33	11	3.70	1.2	
vs.	*vs.*	*vs.*	*vs.*	*vs.*	*vs.*	
(column B)	infected	infected	infected	infected	infected	
One-way ANOVA, post-hoc test (Dunnett)						
P value	<0.0001	<0.0001	0.0003	0.6606	0.7529	
P value summary	****	****	***	ns	ns	
**GC376**						
[μM]	1.00	0.33	0.11	0.04		
vs.	*vs.*	*vs.*	*vs.*	*vs.*		
(column B)	infected	infected	infected	infected		
One-way ANOVA, post-hoc test (Dunnett)						
P value	<0.0001	<0.0001	0.0004	0.0093		
P value summary	****	****	***	**		
**Related to figure 7A: inhibition of IFN-I production**						
**Suramin**						
[μM]	1.1	0.4	0.1	0.04	0.013	0.004
vs.	*vs.*	*vs.*	*vs.*	*vs.*	*vs.*	*vs.*
(column B)	transfected	transfected	transfected	transfected	transfected	transfected
One-way ANOVA, post-hoc test (Dunnett)						
P value	0.1044	0.0012	0.0035	0.2510	0.9881	0.3696
P value summary	ns	**	**	ns	ns	ns
**Related to figure 7B: inhibition of IFN-I signaling**						
**Suramin**						
[μM]	30	10	3.3	1.1	0.4	0.1
vs.	*vs.*	*vs.*	*vs.*	*vs.*	*vs.*	*vs.*
(column B)	transfected	transfected	transfected	transfected	transfected	transfected
One-way ANOVA, post-hoc test (Dunnett)						
P value	0.6178	0.9861	0.8895	>0.9999	0.9998	0.9871
P value summary	ns	ns	ns	ns	ns	ns

Supplementary Table 3, related to figures 6 and 7. Statistical analysis of *in cellulo* experiments Analysis of the statistical significance of Suramin dose-dependent inhibition of SARS-CoV-2 *N* gene transcription, SARS-CoV-2 N-dependent suppression of IFN-I production and SARS-CoV-2 N-dependent inhibition of IFN-I signaling. The number of asterisks proportionally flags the level of statistical significance (ns, not significant).

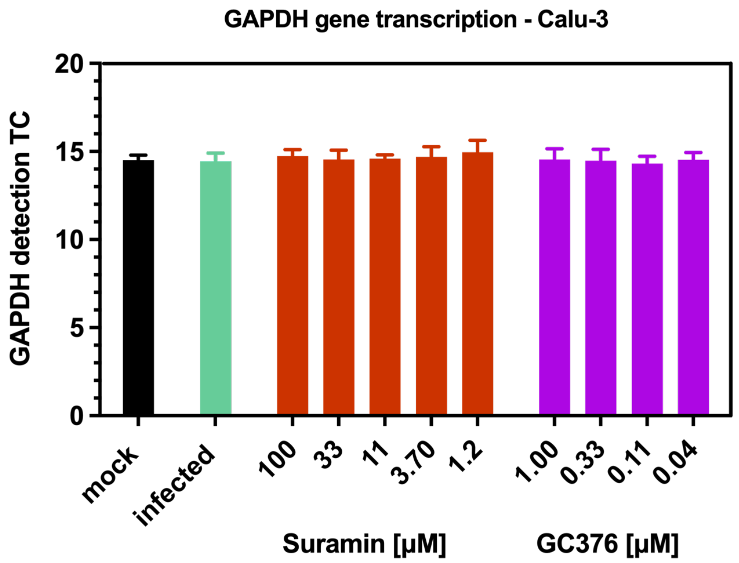

Supplementary figure S5, related to figure 6. Comparative evaluation of GAPDH gene transcription between non infected, untreated infected cells and infected cells treated with Suramin and GC376 Assessment by RT-qPCR of the stable gene expression between samples; GAPDH gene transcription levels were measured in all the samples of the assay performed to test the effect of Suramin and GC376 on SARS-CoV-2 replication in Calu-3 cells (main text, Fig. 6G). Results are expressed as number of TC for the detection of GAPDH transcription. Data are the results of two independent experiments in duplicate.
